# UP150 Project: A Longitudinal Analysis of Active Lifestyles in the Complex Working System

**DOI:** 10.3390/sports12080219

**Published:** 2024-08-14

**Authors:** Gabriele Signorini, Raffaele Scurati, Andrea Bosio, Chiara D’Angelo, Stefano Benedini, Cristina Tringali, Emanuele Magaldi, Marta Rigon, Pietro Luigi Invernizzi

**Affiliations:** 1 Department of Biomedical Sciences for Health, Università degli Studi di Milano, 20133 Milan, Italy; gabriele.signorini@unimi.it (G.S.); stefano.benedini@unimi.it (S.B.); emanuele.magaldi@unimi.it (E.M.); marta.rigon@unimi.it (M.R.); pietro.invernizzi1@unimi.it (P.L.I.); 2 Human Performance Laboratory, Mapei Sport Research Centre, 21057 Olgiate Olona, Italy; andrea.bosio@mapeisport.it; 3 Department of Psychology, Catholic University of the Sacred Heart, 20123 Milan, Italy; chiara.dangelo@unicatt.it; 4 Polispecialistic Clinique San Carlo Srl., 20037 Paderno Dugnano, Italy; 5 Department of Medical Biotechnology and Translational Medicine, Università degli Studi di Milano, 20054 Milan, Italy; cristina.tringali@unimi.it; 6 UCAM Catholic University of Murcia, 30107 Murcia, Spain

**Keywords:** worksite, systems thinking, corporate benefits, metabolic health, psychological health

## Abstract

Workplaces cause employees to adopt sedentary behaviors for most of their daytime, negatively impacting psychophysical health. A new office concept (UP150) was designed to reduce sedentary behaviors at work through architectural changes, proactive technologies, and wellness coaches (education to active lifestyles). The present study examined the effects of the UP150 concept, previously investigated in dedicated workspaces, with a 12-month longitudinal trial in a real worksite environment. Forty-eight desk workers comprised the experimental (EG) and control (CG) groups. All participants worked in the same working environment, having the UP150 features inserted in a usual working environment, but the CG was not allowed to interact with the UP150 specifics. During the experimental year, physical (physical activity, motor efficiency, and anthropometric features), clinical (metabolic parameters and cognitive-capacity-related parameters), and psychological (well-being and discomfort, job social and psychological perceptions, and perceived workload) features were assessed. The prolonged application of the UP150 procedure in a mixed working context for involvement in corporate policies positively affected EG workers’ physical (physical activity and motor efficiency increased, and body fat unchanged), clinical (blood glucose, insulin, and total cholesterol decreased; HDL increased), and psychological (well-being and social support raised; job demand and perceived workload lowered) parameters, confirming the previous studies.

## 1. Introduction

The animal kingdom has a peculiar element, namely, movement, which sets it apart from other living beings [1]. Therefore, humans have been indissolubly linked to the need for movement to procure food or migrate to more favorable conditions for survival [2]. In industrialized countries, human movement is no longer aimed at satisfying survival needs thanks to technological advancement that first helped lighten physically heavier workloads and was later integrated into everyday life [3]. Technology reduced movement and increased daily sedentary behaviors, especially in the desk-worker population [4]. Therefore, the need for movement has shifted towards avoiding the onset of diseases connected to increasingly endemic sedentary behaviors, and maintaining an active physical routine has become the most critical factor in contemporary society’s pursuit of optimal lifelong health. Promoting a cultural approach focused on increasing physical activity through education is necessary to establish healthy movement habits that will last until old age. That means developing individual physical literacy, a multidimensional construct that considers motivation to move, body confidence, physical competence, knowledge, understanding of one’s body, the effects of physical activity on health, and its impact on everyday life to maintain healthy long-life habits [5]. To produce good motor literature acting on individuals’ and group’s affective, physical, behavioral, and social domains is necessary [6]. Hence, disseminating this culture on movement and developing good physical literacy requires considering complex systems that act on multiple aspects of human life.

Systems thinking is a prevailing approach to promoting healthy lifestyles in complex systems [7]. This approach considers the actors involved and how they relate to each other and to the phenomena that influence them to create an interpretative “map” from which to start to modify habits and lifestyles by acting on the facilitators (leverage) or opposers (resistance) to the promotion of health interventions [8]. The analysis of multiple and integrated elements facilitates overcoming interpretative diffraction that would make it challenging to integrate physical activity into the typical workflow, which is generally unfamiliar to this context. That being said, the systems thinking approach to promoting active lifestyles and maintaining good levels of physical literacy could be helpful if used in a complex system such as workplaces that, from a scientific point of view, are gaining increasing interest as environments targeted to engage a significant number of people and reduce sedentary behaviors [9,10].

**Figure 1:**
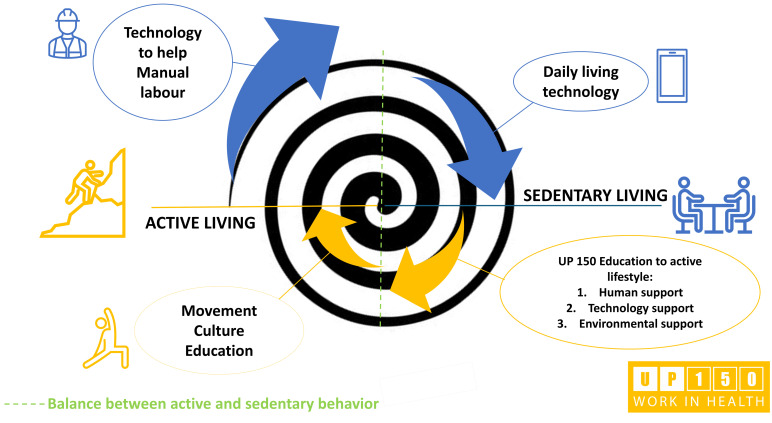
Conceptual spiral of the evolution process of society induced by technological advancement. In blue: technological advancement steps from heavy manual labor (intense physical activity aimed at productivity) to sedentary lifestyles (desk-working). In yellow: steps to recover an adequate amount of workout in the present technologically advanced society (through education on healthy lifestyles using technology and environmental and structural changes to encourage a return to a culture of movement). The dashed green line represents a balance point between active and sedentary behaviors.

Encouraging active lifestyles in the workplace is part of the worksite health promotion policies that aspire to increase workers’ health levels. The most common types of intervention include (i) incorporating specific workout areas located inside the workplace to be used off working hours; (ii) providing incentives for off-site fitness centers to attend after work; and (iii) offering interventions by specialists at the workplace before work, during lunch breaks, after work, or at home [11]. Nevertheless, some barriers come from each of these interventions aimed at promoting physical activity: workers’ spare time (outside of working hours), scheduling (fitting into daily work commitments), fatigue from long working days (too tired at the end of the day to engage in physical activity), and motivation (inadequate perception of the ability to engage in physical activity) [12]. An office concept, the UP150 project, has been designed to address the issues pointed out by the literature. The UP150 project (meaning “proactive office promoting 150 weekly minutes of moderate physical activity at least”) has been proven to induce benefits and behavior changes by including physical activity during the regular workflow based on education to awareness and self-perception [13,14]. It relies on environmental modifications, technological implementation, and support from movement specialists to educate employees on healthy routines and habits to follow during working hours [15]. This form of employee education reduces sedentary behaviors at the workplace and, being inserted into the usual workflow, addresses all workers (including the less active) and promotes and disseminates physical literacy among them as part of a working lifestyle change process (Figure 1).

The principle underlying the new office concept is the education of employees on healthy routines and habits, starting from the workplace and how the workday is approached. Targeting employees at various levels makes it possible to personalize the experience, motivate them, and permanently educate them [16]. In perspective, the UP150 project intends to change the actual corporate welfare visions, implementing a wider taking care of the employees through physical exercise. However, simply implementing tools and technologies to encourage physical activity in the workplace is not enough to change ingrained sedentary habits, as they are likely to be demotivating in the long term. For this reason, into the UP150 concept, the role of a wellness coach has been incorporated alongside modifying work environments, and a dedicated digital app to manage daily physical engagement has been introduced. The wellness coaches operate as a “contagion”, facilitating relationships among colleagues and managers and educating on healthy habits through need-supportive communication, fostering motivational processes linked to the self-determination theory [12,17,18].

**Figure 2:**
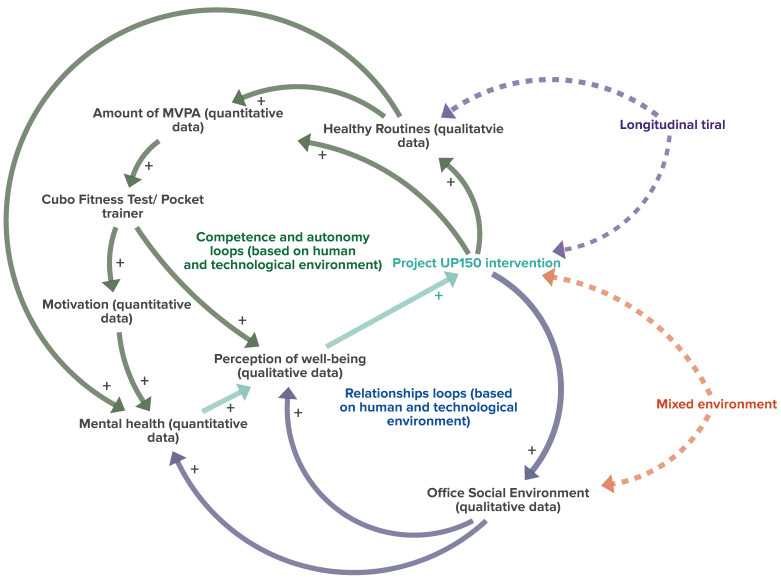
Effects of the UP150 project observed in previous experiments [14] in the office system. Continuous line arrows show interactions between variables (quantitative and qualitative measures). Dashed arrow indicate issues that still need to be investigated. The “+” symbols represent an incremental relationship (increase in the phenomenon) between the connected variables.

Previous studies evaluating the effectiveness of the UP150 concept highlighted that this approach already had positive influences after eight weeks of intervention conducted in a controlled environment (an experimental group placed in a separate setting from the control group) specifically designed to accommodate employees participating in the study (human and technological environments designed to promote active movements, active breaks, and exercises during typical workflow). Beyond increasing the amount of moderate physical activity, levels of physical efficiency, and mental well-being, participants also increased their motivation to adopt active lifestyles and positively changed their work routines [15]. Furthermore, quantitative (questionnaires) and qualitative (semi-structured interviews) analyses based on employees’ perceptions confirmed an improved work environment [14]. From these findings, interactions of the effects of the UP150 concept on the office system can be drawn (Figure 2, solid lines).

The UP150 intervention (Figure 2) clearly positively impacted physical efficiency (measured through the Cubo Fitness Test), boosted motivation (as detected by semi-structured interviews), and enhanced the perception of well-being (by increasing levels of perceived physical competence and autonomy in choosing active exercises—competence- and autonomy-reinforcing loop) [14]. This effect is facilitated by improved interpersonal relationships in the office, as reported by participants during the semi-structured interviews. Moreover, a better social environment, positively influencing mental well-being and increasing the perception of overall well-being, further contributed to increasing commitment to the UP150 concept by enhancing the quality of relationships (relationship-reinforcing loop). From this perspective, the UP150 concept represents a significant leverage point in promoting active lifestyles in the workplace.

The positive results obtained in a controlled environment during the previous 8-week short-term intervention led to the present study’s questioning of whether the UP150 concept can achieve comparable effects over the long term. Therefore, a longitudinal study was conducted in mixed environments of involvement in corporate policies, closer to actual office conditions (both participants involved and not involved in corporate initiatives and policies). The present longitudinal study also aimed to investigate further the effects of the UP150 concept on psychological, job-satisfaction-related, cognitive, and metabolic health clinical factors. We hypothesized that evaluating these additional aspects, integrated into systemic thinking (dashed lines, Figure 2), may boost the promotion of active lifestyles in the corporate setting, confirming their long-term positive effects (as a possible leverage point of the system).

## 2. Materials and Methods

### 2.1. Participants

The sample size for the study was determined by performing a power analysis using the G*Power software (version 3.1.9.4, Universitat Kiel, Germany). The analysis was conducted by selecting the F test mode, ANOVA: repeated measures, within–between interactions. Based on a previous 8-week study [15], an effect size f of 0.25 and a statistical power of 0.95 were chosen. The minimum sample size was then defined as 32 participants. In the current study, 48 participants were recruited from a private company in Milan adhering to the UP150 project. The call for participation was sent by email to workers in agreement with the employer. The following criteria were checked for inclusion in the study: carrying out office work, working at least 6 h per day, being healthy, and having no limiting conditions to physical activity. Participants were randomly assigned to the experimental group (EG, 25 participants) and control group (CG, 23 participants). From the control group, 3 participants dropped out during the study due to work-related reasons (job change or company change), resulting in a final sample of 45 participants, 25 belonging to the experimental group (18 males and 7 females) and 20 to the control group (13 males and 7 females). At the beginning of the experiment, participants of the EG had a mean age of 39.3 ± 11.0 years and a mean BMI of 23.3 ± 3.7 kg/m^2^, while the CG had a mean age of 41.9 ± 10.0 years and a mean BMI of 23.1 ± 3.5 kg/m^2^.

### 2.2. Procedures

The offices of the Progetto CMR company have been modified and implemented with the UP150 concept, consisting of spatial changes, machinery designed to facilitate movement during typical workflow, and workstations equipped with gym tools (active break islands). Among them, meeting rooms were equipped with treadmills and bikes to be used during calls, meetings, and breaks, and micro-physical activities such as hand bikes or steppers served to activate water dispensers, lockers, vending machines, and hand dryers [15,19]. Each participant in the experimental group was assigned a wellness coach whose aim was to motivate physical exercise through supportive communication based on the self-determination theory [20] and to tailor and personalize the participant’s experience. Each participant built up their physical activity with the assistance of the wellness coach to accommodate their needs, in terms of working and free time schedules. Additionally, each worker in the experimental group downloaded a dedicated app (the UP150 app) through which goals were set based on the sub-maximal test for physical efficiency (Cubo Fitness Test). The app was developed to guide employees during their physical activity performed outside and, specifically, in the workplace, during onsite and remote working hours. It records physical activities, assigns scores for each activity (based on perceived effort), and interacts with workspaces (e.g., activating dedicated stations and functions). During the 12-month experimentation period, EG benefited from all components forming the UP150 concept (architectural modifications, app, and wellness coaches), while the control group, even if working in the same environment, did not interact with the concept’s components and was not involved in the project (participants continued with their usual work habits, simulating a group not involved in company policies) [15].

The local ethics committee approved the study (approval nr. 84/20).

**Figure 3:**
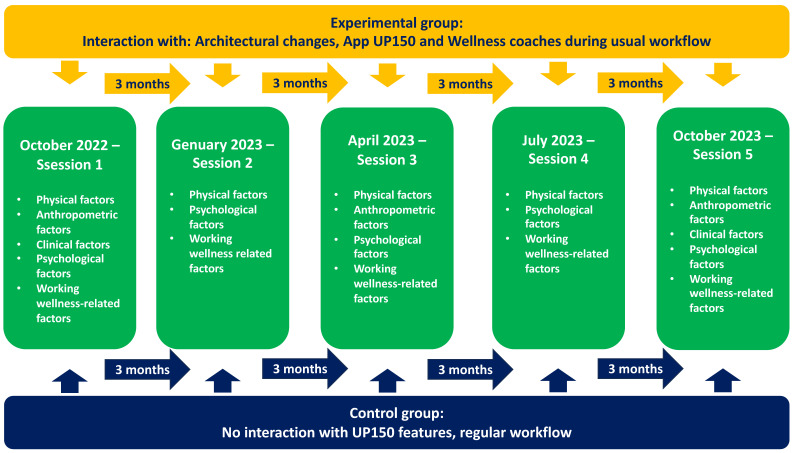
Timeline of the study.

Participants from both groups were familiarized with the tests planned for the experiment. In October 2022 (session #1), all preliminary tests were conducted to measure anthropometric, physical, and psychological features. Measurements were repeated after 6 (in April 2023—session #3) and 12 months (in October 2023—session #5). Additionally, intermediate measurements of physical, psychological, and some other features related to occupational psychological well-being (NASA TLX) were conducted at 3 and 9 months after session #1 (in January and July 2023, sessions #2 and #4, respectively) to monitor and appropriately calibrate the psychophysical workload of the EG participants. Furthermore, blood samples were taken during sessions #1 and #5 to observe clinical factors related to metabolic parameters and cognitive capacity. Figure 3 shows the timeline of the intervention.

Wellness Coach Training

Wellness coaches are professional figures with degrees in sports sciences, specifically trained before the experiment to

(i) educate future participants in understanding and using the TQR recovery assessment [21], the RPE perceived exertion [22], and the Stretch Intensity Scale [23]. These scales are necessary for proposing the sub-maximal motor efficiency tests by the Cubo Fitness Test [13], educating employees on proper body use through self-perception, and for the conscious use of the UP150 app. The technological support given by the app allowed employees to achieve the minimum weekly physical activity score required, adjusting effort intensity appropriately to the situation and one’s psychophysical state.
(ii) administer the questionnaires.
(iii) administer the Cubo Fitness Test.
(iv) promote physical activity using supportive communication (Need Supportive Communication) based on the self-determination theory [24]. The Need Supportive Communication is an empathetic, flexible, and patient communication style that develops autonomy, competence, and relationships among participants [25]. It is considered a valuable and effective communication tool for promoting healthy lifestyles and is positively associated with psychological needs satisfaction and psychophysical health, which aligns with the purposes of the UP150 concept [26].

The wellness coaches educated the employees first on how to properly use all active break stations (with or without machinery), technological devices, and the dedicated UP150 app. Based on previous testing and the analysis of the employee’s specific needs, they helped and guided the workers in performing physical activities by planning and managing exercises and motivating them. The wellness coaches were present in the company two days a week and available remotely (by mail or text messages) during working days.

### 2.3. Measurements

#### 2.3.1. Physical Features

*Physical activity*. The present study utilized Axivity AX3 triaxial accelerometers (Axivity Ltd., Newcastle upon Tyne, UK, 2013) to objectively measure physical activity and sedentary behavior [27]. Participants wore the accelerometers on the non-dominant hand wrist [28] for a full week, from 5:00 PM on Monday to 8:00 AM the following Monday. The accelerometers were configured to capture acceleration within a range of ±16 g at a data collection frequency of 100 Hz [29]. The raw triaxial data were retrieved from the devices and exported using OmGUI software version 1.24 (Axivity Ltd., Newcastle upon Tyne, UK, 2013).

*Motor efficiency*. The Cubo Fitness Test was used to assess the motor efficiency of the participants. It is a test battery consisting of 5 sub-maximal tests based on perceived exertion and muscle stretching aimed at assessing cardiorespiratory (RU, Ruffier test [30]), muscular (PU, push-up and SU, sit-up tests [13,31]), and joint fitness (SM, shoulder mobility [32], and S&R, sit and reach test [33]). The tests returned a motor efficiency index (IME) ranging from 10 to 100 and a weekly physical activity score (WPAS). The IME is the sum of the results obtained in the individual tests normalized for sex and age. A score below 33 points is considered low, between 33 and 66 is considered moderate, and above 66 is considered high. The WPAS is the score to be achieved weekly regarding physical activity and is calculated based on IME, age, and sex. To reach the weekly WPAS, points must be accumulated through physical activities. Each physical activity assigns a score, which is the product of the minutes of activity by a coefficient relative to the perceived intensity on the adapted Borg scale (light perceived activities, with effort values ≤3, have a coefficient of 0.5; moderate activities, effort values between 4 and 6, have a coefficient of 1; vigorous activities, effort values >6, have a coefficient of 2, i.e., a continuous running session of 30 min, perceived as vigorous, accounts/gives for 60 points). The procedures of the Cubo Fitness Test, as well as its validity and reliability, are detailed and confirmed in previous research [15,19,31,34].

*Anthropometric features*. Weight, height, BMI, and body composition were collected. Weight and height were measured using a mechanical scale with an altimeter (Seca 700; Seca North America East, Hanover, MD, USA) with a sensitivity close to 0.1 kg for the scale and 0.1 cm for the altimeter. BMI was calculated using the formula kg/m^2^. Body composition was estimated through skinfold thickness measurements. The measurement was performed using a mechanical skinfold caliper (GIMA skinfold caliper–27320, Gessate, Milano, Italia) calibrated with a sensitivity of 0.2 mm. The body fat percentage was estimated using the Durnin–Womersley formula, commonly used in literature to estimate body composition even in sedentary populations [35,36]. Skinfold measurements were taken at 4 body sites: biceps, triceps, supra iliac, and subscapular. The values, based on age and sex, were inserted into predictive equations for estimating body density. Subsequently, Siri’s formula [37] was used to estimate fat mass. All anthropometric measurements were performed by the same operator, in the same room, with a temperature of 20 °C and humidity of 40%.

#### 2.3.2. Clinical Features

*Metabolic parameters*. Blood samples (22.5) mL were taken sitting via standard antecubital venipuncture. All samples were preserved on ice until plasma or serum centrifugation at 4 °C (within 1.5 h from sampling). Plasma and serum were frozen at −60 °C for later analysis in duplicate. A glucose analyzer measured plasma glucose (Beckman Instruments, Fullerton, CA, USA). Free insulin was dosed via a highly specific two-site monoclonal-antibody-based immunosorbent assay (ELISA; Dako Diagnostics, Cambridgeshire, UK). A commercial ELISA kit served to measure plasma cortisol. A Beckman DXC 700 AU Coulter analyzer detected creatinine, cholesterol, and triglycerides.

*Blood factors related to cognitive capacity*. Levels of BDNF, NGF, and VEGF were measured using 50 mL of plasma for BDNF and 100 mL of plasma for NGF and VEGF through the following ELISA kit: human BDNF (Brain-Derived Neurotrophic Factor) ELISA kit, human NGF (Nerve Growth Factor) ELISA kit, and human VEGF-A (Vascular Endothelial Cell Growth Factor A) ELISA kit (Elabscience, Houston, TX, USA). The reported intra- and inter-assay coefficients of variation were <10%. The reported sensitivity was 18.75 pg/mL for the BDNF ELISA kit, 9.39 pg/mL for the NGF ELISA kit, and 18.75 pg/mL for the VEGF-A ELISA kit. Data were acquired through the microplate reader Victor 2 Wallac 1420 (Perkin Elmer, Waltham, MA, USA).

#### 2.3.3. Psychological Features

*Wellbeing and discomfort*. Because of its reliability and validity in assessing individuals’ self-perception of their overall well-being and discomfort, the Psychological General Well-Being Index (PGWBI) was administered to participants [38,39]. The PGWBI consists of 22 items rated on a 6-point Likert scale that explores 6 dimensions: anxiety, depressive mood, positive well-being, self-control, general health, and vitality. The total sum of all the items is utilized to create an overall general well-being index. In this study, the validated Italian version of the PGWBI was employed [40].

##### Workplace Psychological Wellness Features

*Perceived workload*. The NASA Task Load Index (NASA TLX) was used to investigate the perceived workload related to the employees’ working activity [41,42]. It assesses the workload associated with the previous working week [15,43] considering six domains: mental demand, physical demand, temporal demand, effort, performance, and frustration. A total score is calculated based on each domain’s result and summarizes the overall workload.

*Job social and psychological perceptions*. We used the Job Content Questionnaire to measure job social and psychological characteristics. In the present research, we utilized the adapted and validated Italian version, consisting of 49 questions based on a 4- to 5-point Likert scale and some open questions assessing three main job characteristics: decision latitude, psychological demands, and social support [44,45]. Decision latitude refers to the opportunity to learn new things, the repetitiveness of tasks, the opportunity to utilize one’s competencies, and the level of job organizational leeway; job demands refer to the required physical and psychological work commitment, and social support refers to the working support from coworkers and supervisors [44,45,46].

### 2.4. Statistical Analysis

The normality of the data was verified using the Kolmogorov–Smirnov test and by evaluating the Skewness and Kurtosis [47]. All measurements related to anthropometric factors (weight, height, BMI, and skinfold thickness measurements) and psychological factors (PGWBI and general self-efficacy), along with the Job Content Questionnaire, were analyzed using a 3 × 2 ANOVA (time × group). Measurements related to physical factors (physical activity, Cubo Fitness Test) and NASA TLX were instead analyzed using a 5 × 2 ANOVA (time × group). Post hoc analysis was performed for both tests using unpaired *t*-tests with the Holm–Bonferroni correction. Regarding clinical factors, paired *t*-tests comparing CG and EG sessions #1 and #5 and unpaired *t*-tests comparing EG and CG deltas (session #5–session #1) were conducted. When data normality was not confirmed, group comparisons were conducted using the Mann–Whitney U test (EG vs. CG), while intra-group comparisons were performed using the Friedman test. An alpha value of 0.05 was set for all analyses. The effect size, partial eta squared, calculated for ANOVA, was interpreted using the following cutoffs: 0.01 = small, 0.06 = medium, 0.14 = large [48]. The effect size for *t*-tests (parametric and non-parametric) was calculated using Cohen’s d with the following cutoffs: 0.1 = small, 0.3 = medium, 0.5 = large [49].

## 3. Results

### 3.1. Physical Features

**Figure 4:**
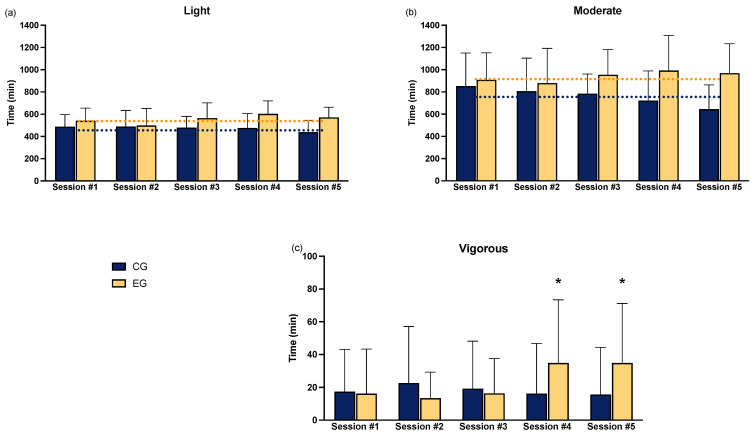
Weekly physical activity (minutes). (**a**) Light activity; (**b**) moderate activity; (**c**) vigorous activity. The dotted lines represent the significant group effect. * = significantly different than CG (*p* < 0.05).

The analysis of the results related to the amount of physical activity measured through the accelerometer showed no significant interactions regarding light physical activity (Figure 4a). However, a group effect was observed (F = 18.505, ηp^2^ = 0.098, *p* < 0.0001). Moderate physical activity (Figure 4b), instead, showed a tendency towards a significant time × group interaction (F = 2.332, ηp^2^ = 0.052, *p* = 0.058) and a significant group effect (F = 14.787, ηp^2^ = 0.099, *p* < 0.0001). For vigorous physical activity (Figure 4c), the non-parametric test revealed differences in sessions #4 (*p* = 0.038, d = 0.30) and #5 (*p* = 0.031, d = 0.31).

**Figure 5:**
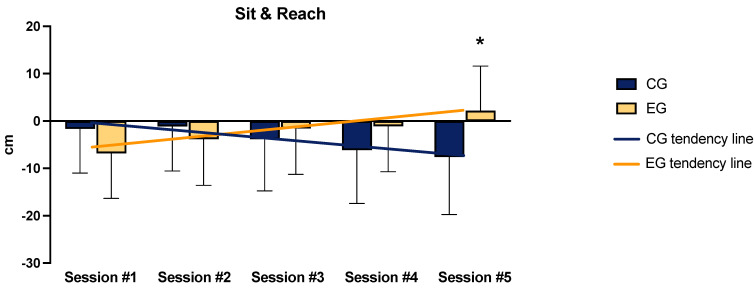
Sit and reach test results. Significantly different than CG: * = *p* < 0.05. The lines represent the time × group effects (interactions).

Regarding the results of the Cubo Fitness Test (Table 1), the RU did not show interaction but a significant group effect (F = 36.226, ηp^2^ = 0.9, *p* = 0.004). The PU and SU tests did not have normally distributed scores; the non-parametric analysis reported significantly higher scores for the EG in sessions #3 (PU: *p* = 0.001, d = 0.48; SU: *p* = 0.041, d = 0.29), #4 (PU: *p* = 0.016, d = 0.35; SU: *p* = 0.022, d = 0.33), and #5 (PU: *p* = 0.001, d = 0.51; SU: *p* = 0.018, d = 0.34) in both tests. In the S&R test (Figure 5), a significant interaction was detected (F = 3.288, ηp^2^ = 0.07, *p* = 0.012), and the post hoc test revealed a difference between groups in session #5 (*p* = 0.007, d = 0.90). No interactions or significant effects were found in the SM test, while a significant group effect was detected for IME (F = 14.718, ηp^2^ = 0.075, *p* < 0.0001). Figure 5 shows the trend of results displaying significant interaction.

The observation of the anthropometric features did not reveal any interaction or effect concerning the measurements related to BMI. However, the body fat percentage calculated by the skinfold measurement showed a significant group effect (F = 19.988, ηp^2^ = 0.146, *p* < 0.0001). The results are reported in Table 2.

### 3.2. Clinical Features

The complete clinical features analysis is reported in Table 3. Specifically, the comparison of the deltas (sessions #5–#1) between the EG and CG showed better significant values of EG glucose values (*p* = 0.021, d = 0.92), insulin (*p* = 0.005, d = 1.15), total cholesterol (*p* = 0.003, d = 1.34), triglycerides (*p* < 0.0001, d = 1.33), and HDL (*p* = 0.029, d = 0.88). Regarding parameters related to cognitive-capacity-related parameters, significant differences were found in favor of the EG only in the VEFG delta values (*p* < 0.0001, d = 1.6).

### 3.3. Psychological Features

**Figure 6:**
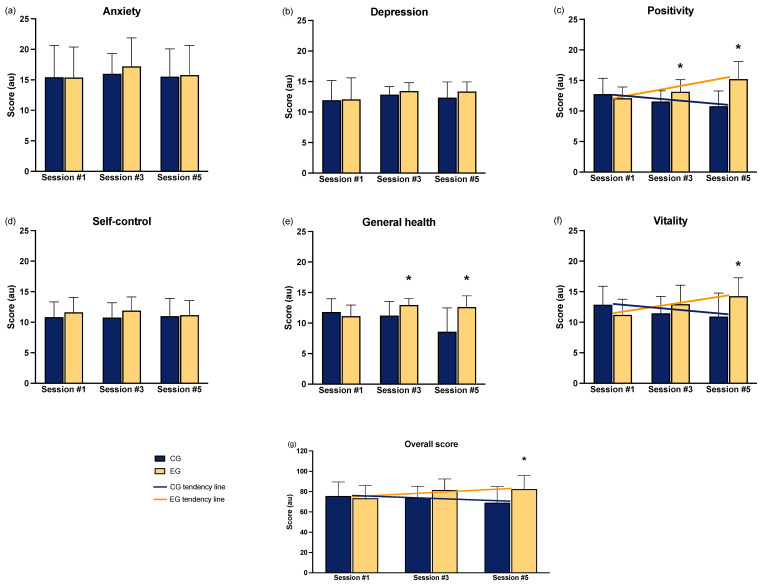
Results of the PGWBI questionnaire for each dimension: (**a**) anxiety; (**b**) depression; (**c**) positivity; (**d**) self-control; (**e**) general health; (**f**) vitality. Panel (**g**) shows the overall score. Significantly different than CG * = *p* < 0.05. The lines represent the time × group effects (interactions).

In Figure 6, the results of the PGWBI questionnaire are presented. Significant interactions were found in positivity (F = 8.505, ηp^2^ = 0.145, *p* < 0.0001), vitality (F = 5.539, ηp^2^ = 0.101, *p* = 0.005), and overall score (F = 3.415, ηp^2^ = 0.065, *p* = 0.037), indicating a positive trend in favor of the EG. Post hoc analysis showed significant variations between the EG and CG in positivity in sessions #3 (*p* = 0.025, d = 0.86) and #5 (*p* = 0.017, d = 1.64), and in vitality (*p* = 0.017, d = 0.96) and the total score (*p* = 0.017, d = 0.90) in session #5. The general health category was analyzed using a non-parametric test due to non-normal distribution, which revealed significant differences in favor of the EG in sessions #3 (*p* = 0.021, d = 0.34) and #5 (*p* = 0.001, d = 0.48).

#### Workplace Psychological Wellness Features

A significant time effect (F = 13.282, ηp^2^ = 0.186, *p* < 0.0001) was observed in the decision latitude of the Job Content Questionnaire (Table 4), even if without a significant interaction. Job demand showed a significant interaction (F = 10.985, ηp^2^ = 0.158, *p* < 0.0001) with post hoc differences between the EG and CG in session #5 (*p* = 0.017, d = 1.28). Values related to social support were analyzed using a non-parametric analysis and showed significant differences in favor of the EG in session #5 (*p* < 0.0001, d = 0.56).

The results related to workload (Table 5) indicate a significant interaction in mental demand (F = 2.794, ηp^2^ = 0.06, *p* = 0.028). However, the post hoc analysis did not reveal significant differences between groups, even if data values of the EG seemed to have a decreasing trend, contrary to the CG results that increase, which explains the observed interaction. No interactions or significant effects were found for the other NASA TLX parameters except for the effort, having a significant group effect (F = 6.383, ηp^2^ = 0.012, *p* = 0.035).

## 4. Discussion

This study investigated the effects of the UP150 project in a 12-month longitudinal trial in a mixed environment (i.e., involving engagement with company policies related to the UP150 project). From a system thinking perspective, the study evaluated how the UP150 concept impacts employees’ physical, clinical, psychological, and work-related well-being factors thanks to actions promoting education to move.

### 4.1. Physical Features

**Figure 7:**
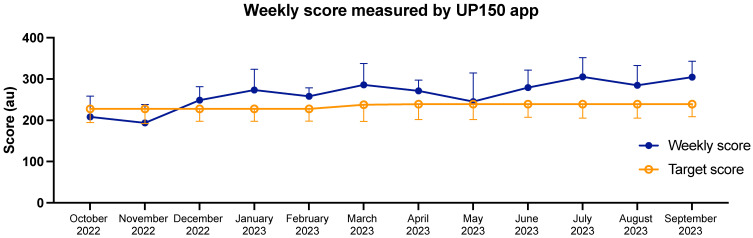
The trend of weekly scores of the experimental group participants throughout the first year of intervention, as recorded by the UP150 app. The performed weekly score is the average of the weekly scores achieved by the participants during each month. The target weekly score represents the average of the target scores EG should reach weekly, determined by the baseline testing with the Cubo Fitness Test.

*Physical activity*. During this longitudinal study, experimental group participants gradually increased the amount of physical activity performed. The analysis revealed that EG performed more light and moderate physical activity during the experimentation year. Moreover, in the last two experimental sessions (#4 and #5), EG resulted in more vigorous physical activity than CG. As expected, UP150 effectively promoted light and moderate-intensity physical activity, consistent with previous studies [15] and coherently with other workplace interventions [50]. As per the protocol, wellness coaches are tasked with promoting active lifestyles using tools such as the UP150 app and Cubo Fitness Test, which, through perceived exertion, allow employees to achieve the minimum amount of physical activity recommended by the WHO (150 min/week of moderate physical activity or 75 min/week of vigorous physical activity) [51]. After the second month, the results indicate that participants exceeded the expected weekly score and maintained a steady trend throughout the experiment (Figure 7).

All exercises in the office recommended by the wellness coaches were to be performed with a perception of effort from light to moderate to avoid conflicting with work demands (excessive sweating, excessive vigor in a formal environment, or risk of injuries). Light activities increased alongside moderate activities, likely due to the gradual incorporation of active micro-breaks into the new resulting work routines, allowing them to integrate into the workday more activity in less time (e.g., completing joint mobility light activities in just one to three minutes). Furthermore, wellness coaches supplied recommendations and motivation for physical activity and healthy lifestyles outside working hours through need-supportive communication by digital technology, further recommending vigorous activities.

The trend of improvements could indicate that light and moderate activities, primarily promoted by the coaches and practicable in the office, first impacted EG employees. Instead, the employees probably autonomously integrated vigorous activities outside the workplace in the last months of the trial. In summary, the overall trend of the results related to the amount of physical activity suggests that the motivation to move originated in the workplace thanks to direct involvement by the wellness coach and later developed into an autonomous intention to exercise not only within but also outside the workplace (self-dependent activities fostered by the wellness coaches) [52,53]. The Cubo Fitness Test’s results confirm this trend: although the significant interaction suggests a constant improvement in parameters related to joint mobility, significant improvements compared to the CG first appeared in muscular fitness, starting from the third measurement session (at mid-experimentation). Afterward, significant improvements were also found in cardiorespiratory fitness. Finally, summing up all results, a general improvement in motor efficiency (IME) was observed, leading the EG to outperform the CG.

It is still being determined which fitness exercises the employees performed the most frequently. However, this improvement is linked to increased exercise performed by EG participants, consistent with the literature [54].

*Anthropometric features*. All participants started from an average condition of normal weight and body fat percentages within the recommended ranges for health [55,56]. Despite this, the intervention had positive effects on body composition parameters. The body fat percentage trend observation shows that initial values were comparable at mid-experimentation and were slightly reduced at the end of experimentation in the EG. Differently, the CG slightly increased their body fat percentage, producing significant differences between the groups. This effect is expected if we consider the increase in the weekly physical activity performed by the EG, predominantly at moderate intensity. As highlighted by Winters, et al. [57], weight control can be facilitated by reducing the time spent in sedentary activities and increasing moderate to vigorous physical activity (MVPA). According to this study, 30 more minutes per week of MVPA reduced total body fat percentage by 1.3% (participants had a BMI of 27 kg/m^2^ or higher). In our case (with normal-weight participants), an average increase of 78 min per week of MVPA (+60 min of moderate activity and +18 min of vigorous activity) reduced body fat by 0.8%.

### 4.2. Clinical Features

Consistently with what has been previously highlighted, the delta analysis showed that after the experimentation, EG participants had significantly higher improvements in metabolic clinical parameters compared to the CG. Specifically, the EG improved blood glucose, total circulating cholesterol, insulin, triglycerides, and HDL levels. These findings and improvements of all EG parameters between sessions #1 and #5 confirm that engaging in a minimum weekly physical activity can positively impact health and certain metabolic parameters associated with metabolic syndrome [51]. The study data also suggest that these positive effects can be achieved even with short active breaks of less than 10 min, as previously found in the literature [58].

Regarding blood factors related to cognitive capacity, the increase in moderate-intensity physical activity and its spreading throughout the day may have positively influenced EG’s VEGF, as reported by the literature [59,60]. However, there were no positive effects on the other two parameters measured (BDNF and NGF). We hypothesize that this is possibly due to the short duration of active breaks, which resulted in insufficient to modify them. Nevertheless, vascular development could still have brought benefits to the brain, as it is associated with better oxygen diffusion in tissues, which helps prevent neurodegenerative diseases like Alzheimer’s disease [61].

### 4.3. Psychological Features

As in a previous 8-week study [15], the longitudinal intervention brought psychological and mental health benefits. From the PGWBI, it is noted that from session #3 (6 months from the beginning of the intervention), general health and positivity are higher in the EG than in CG, and, at the end of the intervention, the same resulted in vitality. Two explanations can be adduced: the coping aspects of the low-intensity physical exercise performed in pauses during the workflow and the communicative procedures used by wellness coaches based on self-determination theory (need-supportive communication) [25]. Several studies have shown that low-intensity exercise can reduce or help manage stress [62,63] and increase vitality [64]. From a cognitive point of view, vitality carries an increased sense of well-being, lower levels of mental fatigue, and greater resilience and perseverance. Research conducted in the work environment has found that augmented positivity and vitality can increase productivity and involvement, reducing the risk of burnout [65]. In addition, the need-supportive communication based on the self-determination theory used by wellness coaches has been proven effective in increasing mental well-being, even in the workplace, by acting on the sense of autonomy, competence, and mutually satisfying relationships [52].

#### Workplace Psychological Wellness Features

The mental well-being of employees is also reflected in factors related to working well-being. The analysis of the Job Content Questionnaire outcomes reveals a lower perception of the workload of the EG employees compared to the control. Moreover, at the end of the longitudinal intervention, employees who took an active part in the UP150 concept reported higher levels of social support, a value linked to collaboration in the workplace. The reduced perception of workload could originate from the change in the working paradigm experienced by employees who moved from a standard model providing a few coded macro-breaks (for example, lunch or coffee break) to a new model (the UP150) inserting active micro-breaks spread throughout the day (1 to 5 min about every 60 min). This would have positively affected their perception of well-being, in line with Radwan and colleagues’ findings [66]. The gradual reduction in mental demand for EG, as seen from the NASA TLX outcomes, further supports this occurrence. In the previous UP150 study, this parameter also improved after intervention, confirming significantly lower levels of mental demand of participants experiencing intervention. Thanks to the consolidation of the active routines in the workplace, this perceptive modification could have led employees to report less work effort in the NASA TLX questionnaire.

The improved social support parameter could originate from the wellness coach’s support based on self-determination theory. Indeed, from the semi-structured interview conducted in the previous 8-week UP150 study, employees already reported an advance in the relational climate in the working environment favored by wellness coaches through active group breaks or moments of informative workshops [14,67]. In line with the literature [68], the effectiveness of the UP150 concept in creating meaningful relationships with the social environment and a sense of acceptance within the working environment through need-supportive communication is confirmed.

### 4.4. Summary

Although several outcomes of this study confirm the results from a previous study [15], the two studies differ in the temporal span necessary to detect changes. In this research, noticing significant differences between EG and CG required, for many outcomes, a minimum of six months of intervention, while, in Invernizzi’s study, the first results in physical (physical efficiency and physical activity) and psychological features (health status and workload) appeared after two months. This discrepancy can be explained by the different setting aspects in which the two experiments were carried out. In the first study, the worksite was specifically created to host the experiment, and only the experimental participants worked in it; in the present study, both the EG and CG participants worked together in the same office that was implemented with architectural changes, but in which the control did not follow the UP150 concept. This mixed environment, corresponding to the actual condition of reality where the concept could be inserted in the future, could have generated more resistance to changes because of social conditioning [69]. In Figure 7, the inertia in achieving the weekly score in the first two months of intervention can be easily noticed. In mixed environments about motivation to exercise, active breaks may be unusual and not understood if not perceived as disrupting and interrupting the work task by those not involved in the specific UP150 project. The preservation mechanism of the social self (i.e., self-image within the group, social status, social esteem, and group acceptance) leads the individual to preserve himself from situations the group may not accept, thus leading to sensations of shame [69]. From a system thinking perspective, the presence of mixed environments for involvement in physical activity could be a point of resistance that leads to increased time needed to educate employees to adopt healthy lifestyles and receive the resulting benefits.

**Figure 8:**
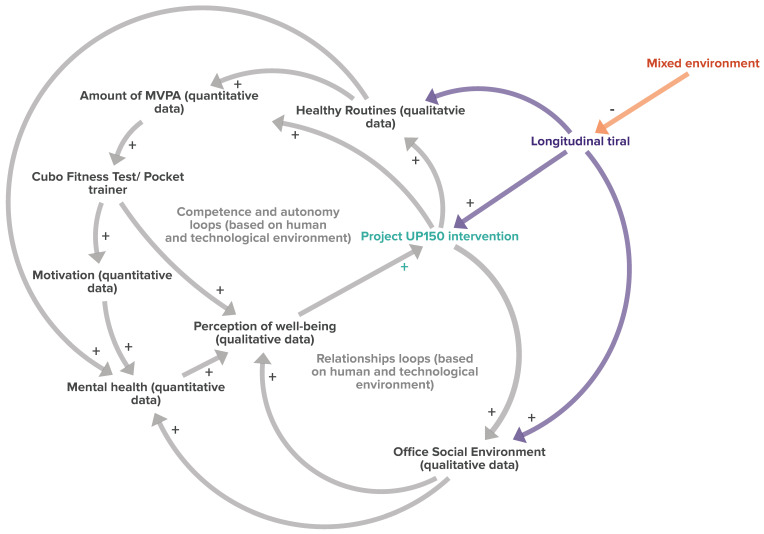
Interactions of variables of the mixed environment of the longitudinal study and the UP150 approach features (purple arrows and words). Grey lines and words: results from the previous study on the UP150 approach. Continuous lines: interactions between variables (from measurements and semi-structured interviews). The positive or negative influence between variables is evidenced by symbols + and -. The green and orange words indicate the influence effects of the present study.

Figure 8 shows how a mixed environment and a longitudinal intervention fit into the UP150 system thinking. The mixed environment (the limited participation of employees in company policies) slows down the occurrence of potential positive effects, which requires an initial period of adaptation to reach regime levels (in our case, two months, as in Figure 7). Despite this, the UP150 concept benefited from the long duration of the intervention, preserving motivation to exercise and establishing ongoing improvements throughout the twelve months of protocol supply.

### 4.5. Limitations

Some limitations in this study may have affected the results: the number of participants and the need for more information on complementary factors such as workers’ job performance and sickness absences. Even if the sample resulted adequately for this study design, it was considered insufficient for an explanatory statement of the gender-specific and age-specific impact of the UP150 concept. Concerning possible effects of the UP150 concept on employee job performance or reduction of days of absence (that are presumed to be possible favorable outputs of the intervention), privacy issues and company policies do not permit the collection of job-related personal information, which in future studies would complete the analysis of benefits for workers and employers.

Lastly, we did not analyze the specifics of the physical activities that the experimental participants performed during the workflow and in their spare time. However, as the protocol intended to differentiate physical engagement to adapt to all the participants’ conditions and needs, we considered only the overall amount and intensity of physical activity (measured by accelerometers and defined by the weekly score returned by the UP150 app) as an indicator of physical exercising, and there was no need to collect detailed exercise composition.

## 5. Conclusions

The prolonged application of the UP150 procedure in a mixed working context for involvement in corporate policies, such as in the present longitudinal intervention, confirmed the preliminary result of the previous study and positively affected workers’ physical and psychological well-being. Moreover, the present study brought new important insights highlighting the positive effect of the concept on clinical well-being (glucose, insulin, triglycerides, total cholesterol, and HDL). However, unlike the previous studies, more time to detect the effects is required (at least six months), possibly because the mixed environment consequences alter the UP150 routines of the workers involved as an experimental group. Within a broader vision, education for the movement, prolonged over time, is a leverage point for the whole system. On the contrary, a mixed environment with both people engaged and non-engaged in the UP150 concept, in a particular context such as that of the office, could be a point of resistance to changing approach and move during the workday, denoting how the evolution of the working culture must necessarily start from shared and encouraged company policies. Ultimately, the UP150 concept, thanks to technology closely assisted by the education in exercising as provided by wellness coaches through supportive communication, maintains its effects over time and is effective in building and consolidating virtuous routines for health.

## Figures and Tables

**Table 1:** Physical efficiency (Cubo Fitness Test).

Time Point	Group	RU (au) $	PU (au)	SU (au)	SM (cm)	S&R (cm) §	IME (au) $
Session #1	EG	7.8 ± 2.9	3.6 ± 1.6	6.7 ± 3.8	53.5 ± 8.4	−1.6 ± 9.3	51.6 ± 11.0
	CG	9.4 ± 2.9	3.3 ± 1.8	5.4 ± 2.4	50.5 ± 12.1	−6.8 ± 9.5	48.1 ± 9.3
Session #2	EG	7.1 ± 4.2	4.7 ± 3.1	7.0 ± 4.2	56.3 ± 8.4	−3.8 ± 9.8	48.5 ± 15.9
	CG	9.0 ± 2.9	3.1 ± 1.3	5.6 ± 3.6	51.3 ± 10.8	−1.1 ± 9.4	46.6 ± 10.4
Session #3	EG	6.8 ± 3.3	5.4 ± 2.6 *	8.0 ± 4.1 *	52.9 ± 9.2	−1.5 ± 9.7	56.3 ± 11.8
	CG	8.1 ± 3.1	3.1 ± 1.3	5.4 ± 2.0	51.3 ± 11.0	−3.8 ± 10.9	49.8 ± 10.0
Session #4	EG	5.8 ± 2.1	5.4 ± 2.6 *	7.7 ± 4.1 *	53.5 ± 9.2	−1.1 ± 9.6	57.9 ± 11.3
	CG	8.5 ± 3.3	3.2 ± 1.3	4.9 ± 1.9	50.7 ± 11.1	−6.2 ± 11.2	49.0 ± 8.0
Session #5	EG	6.1 ± 2.7	5.4 ± 3.6 *	8.3 ± 4.1 *	53.0 ± 8.8	2.2 ± 9.4 *	57.1 ± 10.4
	CG	9.3 ± 3.6	3.2 ± 1.3	5.2 ± 2.1	52.4 ± 12.4	−7.6 ± 12.2	46.4 ± 10.6

EG = experimental group, CG = control group, RU = Ruffier test, PU = push-up test, SU = sit-up test, SM = shoulder mobility, S&R = sit and reach test, IME = index of motor efficiency. Significantly different than CG: * = *p* < 0.05. Significant interaction (time × group): § = *p* < 0.05. Significant group effect: $ = *p* < 0.05.

**Table 2:** Body composition.

	Session #1		Session #3		Session #5	
	EG	CG	EG	CG	EG	CG
BMI (Kg/m^2^)	23.3 ± 3.7	23.1 ± 3.5	23.4 ± 3.7	23.3 ± 3.5	23.2 ± 3.8	23.1 ± 3.5
% Body Fat $	25.5 ± 6.0	27.2 ± 6.5	25.6 ± 6.2	28.4 ± 7.0	24.7 ± 6.1	27.4 ± 7.4

EG = experimental group, CG = control group. Significant group effect: $ = *p* < 0.05.

**Table 3:** Clinical features.

	EG		CG		Delta (#5–#1)	
	Session #1	Session #5	Session #1	Session #5	EG	CG
Creatinine (mg/dL)	0.92 ± 0.20	0.84 ± 0.22 *	0.86 ± 0.12	0.77 ± 0.16 *	−0.09 ± 0.06	−0.08 ± 0.08
Glucose (mg/dL)	83.4 ± 7.65	75.20 ± 6.32 *	81.92 ± 8.95	83.77 ± 8.17	−6.53 ± 5.94 §	−0.08 ± 7.90
Insulin (µU/mL)	5.45 ± 1.85	4.22 ± 1.22 *	6.80 ± 7.68	7.18 ± 8.08	−1.11 ± 1.04 §	0.23 ± 1.27
Total cholesterol (mg/dL)	186.67 ± 27.05	172.07 ± 21.18 *	206.92 ± 56.31	211.85 ± 61.10	−14.87 ± 7.80 §	5.23 ± 19.63
Triglycerides (mg/dL)	82.93 ± 29.65	76.67 ± 25.46 *	86.00 ± 47.89	110.62 ± 47.27 *	−1.93 ± 12.92 §	19.62± 8.90
HDL (mg/dL)	60.00 ± 11.64	64.33 ± 11.99 *	67.38 ± 16.33	62.38 ± 14.39 *	2.80 ± 7.02 §	−3.23 ± 6.67
Cortisol (mcg/dL)	15.71 ± 9.88	12.50 ± 8.03 *	11.82 ± 2.61	14.50 ± 6.52	−2.48 ± 3.70	1.83 ± 7.84
BDNF (pg/mL)	8495.7 ± 2081.9	7778.9 ± 2818.0	8880.6 ± 2603.2	7412.3 ± 2818.8 *	−716.8 ± 2242.3	−1468.3 ± 2261.5
VEGF (pg/mL)	162.2 ± 118.9	252.2 ± 182.5 *	223.0 ± 98.7	139.1 ± 73.8 *	90.0 ± 128.4 §	−83.8 ± 84.0
NGF (pg/mL)	153.1 ± 261.2	74.9 ± 146.6	143.4 ± 234.0	182.1 ± 289.6	−78.2 ± 198.2	38.7 ± 203.5

EG = experimental group, CG = control group. HDL = high-density lipoproteins, BDNF = brain-derived neurotrophic factor, VEGF = vascular endothelial growth factor, NGF = nerve growth factor. * = significantly different than session #1 in paired *t*-test (*p* < 0.05); § = significantly different than CG in unpaired *t*-test (*p* < 0.05).

**Table 4:** The Job Content Questionnaire’s results.

	Session #1		Session #3		Session #5	
	EG	CG	EG	CG	EG	CG
Decision latitude (au) ⸸	77.5 ± 7.5	73.9 ± 10.6	76.8 ± 8.1	72.2 ± 6.0	61.2 ± 25.4	58.9 ± 19.8
Job demand (au) §	36.1 ± 3.7	34.5 ± 2.4	32.3 ± 6.0	34.4 ± 3.6	21.0 ± 12.8 *	33.5 ± 5.1
Social support (au)	19.7 ± 5.9	23.1 ± 5.2	23.7 ± 3.9	25.4 ± 4.8	25.7 ± 3.3 *	20.2 ± 4.4

Time points: session #1: October 2022; session #3: April 2023; session #5: October 2023. EG = experimental group, CG = control group. Significantly different than CG: * = *p* < 0.05. Time × group interaction: § = *p* < 0.05. Significant time effect: ⸸ = *p* < 0.05.

**Table 5:** Workload (NASA TLX).

Time Point	Group	MD (au) §	PD (au)	TD (au)	PE (au)	EF (au) $	FR (au)	WS (au)
Session #1	EG	74.0 ± 17.7	11.5 ± 12.6	44.7 ± 24.7	34.7 ± 18.3	26.7 ± 18.0	6.9 ± 10.6	13.2 ± 2.8
	CG	61.9 ± 23.9	7.5 ± 8.0	43.2 ± 21.2	31.1 ± 17.7	29.4 ± 20.7	4.6 ± 7.4	11.8 ± 3.5
Session #2	EG	62.8 ± 19.8	9.1 ± 10.6	52.4 ± 23.4	35.8 ± 26.7	25.6 ± 18.6	14.4 ± 24.9	13.3 ± 3.4
	CG	55.1 ± 15.6	11.7 ± 11.2	44.0 ±23.7	28.5 ± 22.7	27.9 ± 15.5	13.1 ± 24.1	12.0 ± 2.0
Session #3	EG	65.8 ± 22.9	11.0 ± 16.1	48.9 ± 18.9	23.3 ± 13.9	30.3 ± 20.0	13.6 ± 23.1	12.9 ± 3.2
	CG	60.6 ± 27.4	11.2 ± 15.8	43.6 ± 21.7	21.2 ± 12.8	26.1 ± 20.4	7.3 ± 9.5	11.3 ± 2.0
Session #4	EG	53.3 ± 22.9	8.2 ± 10.0	50.3 ± 18.7	34.7 ± 21.9	22.3 ± 15.9	12.3 ± 16.6	12.1 ± 3.3
	CG	67.2 ± 23.2	6.1 ± 10.1	46.3 ± 27.0	22.3 ± 13.6	29.2 ± 23.8	19.7 ± 25.9	12.8 ± 2.9
Session #5	EG	59.2 ± 16.9	7.3 ± 9.9	51.2 ± 19.7	23.1 ± 14.0	28.6 ± 21.4	15.8 ± 18.1	12.3 ± 2.3
	CG	73.3 ± 25.9	16.6 ± 20.9	48.6 ± 25.7	22.8 ± 10.0	36.8 ± 27.1	8.2 ± 15.5	13.7 ± 2.6

EG = experimental group, CG = control group, MD = mental demand, PD = physical demand, TD = temporal demand, PE = performance, EF = effort, FR = frustration, WS = weighted sum. Significant interaction (time × group): § = *p* < 0.05. Significant group effect: $ = *p* < 0.05.

## Data Availability

The data presented in this study are available on request from the first author due to privacy reasons.
